# A randomised controlled study shows supplementation of overweight and obese adults with lactobacilli and bifidobacteria reduces bodyweight and improves well-being

**DOI:** 10.1038/s41598-020-60991-7

**Published:** 2020-03-06

**Authors:** D. R. Michael, A. A. Jack, G. Masetti, T. S. Davies, K. E. Loxley, J. Kerry-Smith, J. F. Plummer, J. R. Marchesi, B. H. Mullish, J. A. K. McDonald, T. R. Hughes, D. Wang, I. Garaiova, Z. Paduchová, J. Muchová, M. A. Good, S. F. Plummer

**Affiliations:** 1 Cultech Limited, Unit 2 Christchurch Road, Baglan Industrial Park, Port Talbot, United Kingdom; 2 School of Biosciences, Cardiff University, Cardiff, United Kingdom; 3 Division of Digestive Diseases, Department of Metabolism, Digestion and Reproduction, Faculty of Medicine, Imperial College London, London, United Kingdom; 4 MRC Centre for Molecular Bacteriology and Infection, Imperial College London, London, United Kingdom; 5 Systems Immunity Research Institute, Henry Welcome Building, Cardiff University, Cardiff, United Kingdom; 6 Department of Clinical Sciences, Liverpool School of Tropical Medicine, Liverpool, United Kingdom; 7 Institute of Medical Chemistry, Biochemistry and Clinical Biochemistry, Faculty of Medicine, Comenius University, Bratislava, Slovakia; 8 School of Psychology, Tower building, Cardiff University, Cardiff, United Kingdom

**Keywords:** Nutritional supplements, Nutrition

## Abstract

In an exploratory, block-randomised, parallel, double-blind, single-centre, placebo-controlled superiority study (ISRCTN12562026, funded by Cultech Ltd), 220 Bulgarian participants (30 to 65 years old) with BMI 25–34.9 kg/m^2^ received Lab4P probiotic (50 billion/day) or a matched placebo for 6 months. Participants maintained their normal diet and lifestyle. Primary outcomes were changes in body weight, BMI, waist circumference (WC), waist-to-height ratio (WtHR), blood pressure and plasma lipids. Secondary outcomes were changes in plasma C-reactive protein (CRP), the diversity of the faecal microbiota, quality of life (QoL) assessments and the incidence of upper respiratory tract infection (URTI). Significant between group decreases in body weight (1.3 kg, *p* < 0.0001), BMI (0.045 kg/m^2^, *p* < 0.0001), WC (0.94 cm, *p* < 0.0001) and WtHR (0.006, *p* < 0.0001) were in favour of the probiotic. Stratification identified greater body weight reductions in overweight subjects (1.88%, *p* < 0.0001) and in females (1.62%, *p* = 0.0005). Greatest weight losses were among probiotic hypercholesterolaemic participants (−2.5%, *p* < 0.0001) alongside a significant between group reduction in small dense LDL-cholesterol (0.2 mmol/L, *p* = 0.0241). Improvements in QoL and the incidence rate ratio of URTI (0.60, *p* < 0.0001) were recorded for the probiotic group. No adverse events were recorded. Six months supplementation with Lab4P probiotic resulted in significant weight reduction and improved small dense low-density lipoprotein-cholesterol (sdLDL-C) profiles, QoL and URTI incidence outcomes in overweight/obese individuals.

## Introduction

World Health Organisation (WHO) global estimates indicate that 39% of adults are overweight and 13% are obese and trends suggest that levels will continue to rise as a result of current dietary habits and sedentary lifestyles^1^. The burden of obesity on primary healthcare providers is substantial and it is estimated that, in England alone in 2013, excess body weight in women cost £2.2 billion in consultations and £1.9 billion for prescription medications^2^. One of the consequences of obesity is the increased incidence of Metabolic Syndrome (MetS) - an umbrella term used for a cluster of interrelated metabolic conditions linked with obesity including hypercholesterolaemia, hyperglycaemia and hypertension and associated with diabetes, cardiovascular disease (CVD) and dementia^3,4^. To prevent the development of MetS, adoption of a healthy diet and active lifestyle to avoid excessive weight gain is probably the most accessible strategy, but the ongoing rise in global obesity suggests that such lifestyle modifications are difficult to adopt by the general population^1^. The problem is compounded by the difficulty of maintaining any weight losses - more than half of the weight lost by an individual is regained within 2 years and more than three-quarters is regained within 5 years^5^.

There is a clear need for other approaches to aid weight loss and/or prevent weight gain/re-gain and one strategy is to target the gut microbiome. The trillions of microorganisms residing in the gastrointestinal tract contribute to the gut microbiome which plays a critical role in host metabolism through a diverse repertoire of functions including the modification and/or liberation of dietary nutrients, immuno-modulation and the regulation of bile acid metabolism^4^. Stability of the gut microbiota is being closely linked with well-being and there is growing evidence that microbial imbalance may be linked with the pathogenesis of obesity^6^ and other metabolic diseases including CVD^7^. Modulation of the composition/functionality/stability of the gut microbiota is being seen as an approach to support the prevention of the obesity and MetS development march^8^. Manipulation of the microbiota can be achieved through dietary supplementation with probiotic bacteria (defined by WHO as “live microorganisms that, when administered in adequate amounts, confer a health benefit on the host”^9^). Probiotic supplementation is receiving much attention due to a growing body of evidence demonstrating safety and beneficial impacts on many aspects of human health including metabolism and immunity^10,11^. The Lab4P consortium of probiotics (composed of *Lactobacillus acidophilus*, *Bifidobacterium bifidum, Bifidobacterium animalis* subsp. *lactis* and *Lactobacillus plantarum*) has shown cholesterol lowering capability^12^ and an ability to suppress diet induced weight gain in mice^13^.

The aim of the current study was to assess the hypothesis that Lab4P daily supplementation over a period of 6 months in a healthy overweight/obese free-living human cohort would provide beneficial effects on body weight and well-being. Outcomes included changes in anthropometric measurements, plasma lipids and plasma C-reactive protein (CRP). Participant perceived quality of life (QoL) and the incidence of upper respiratory tract infection (URTI) were monitored as indicators of general well-being with stratification of the study population to identify subgroups of participants benefitting most from probiotic supplementation.

## Methods

### Study approval

This study was conducted according to the principles of the Declaration of Helsinki and with approval from the Ethical Committee of Comac Medical, Sofia, Bulgaria (Reference: #127/20.06.2017). The study design has been deposited in the ISRCTN registry (ISRCTN12562026 Registration date: 12.03.2019).

### Study design

This was a single-centre, double-blind, randomised and placebo-controlled superiority study with equal allocation of participants between two parallel study groups. As an exploratory study, there was no formal sample size calculation.

### Recruitment and randomisation

The study was performed by the trials company Comac Medical. Adults aged 30–65 were recruited at the trials facility (Sofia, Bulgaria) between 17/07/17 and 26/07/17. The included participants had a waist circumference > 89 cm (women) or > 100 cm (men); a body mass index (BMI, kg/m^2^) between 25 and 34.9; receiving no statins or on stabilised statin therapy for at least 3 months and were willing to provide blood samples. Participants were not considered if they were undergoing immunodeficiency/immunosuppressive therapy; had diagnosed diabetes; pregnant or planning pregnancy; had history of ischemic heart disease, heart failure, prolonged QTc interval, rhythm, conduction disorders or any other cardiovascular disease deemed by the investigator as a risk for the participation in the study; had severe systemic disease (cancer, dementia, advanced organ failure); or had experienced significant unexplained weight loss in the previous 3 months.

All participants entering the study provided written informed consent and received a financial incentive as stipulated by the ethics committee. Eligible participants were sequentially assigned an order number and allocated to one of 2 arms of the study in a 1:1 ratio according to a computer-generated random sequence using permuted block randomisation with a block-size of four. The randomisation scheme was generated by an independent statistician using SAS PROC PLAN (SAS v9.4) and the study product was randomised before arrival at the trial site. The allocation sequence was not available to any member of the research team until databases had been completed and locked but was held at the trial site in tamper-proof sealed envelopes in case of emergency.

### Study product

The active product (Lab4P) comprised *Lactobacillus acidophilus* CUL60 (NCIMB 30157), *Lactobacillus acidophilus* CUL21 (NCIMB 30156), *Lactobacillus plantarum* CUL66 (NCIMB 30280) *Bifidobacterium bifidum* CUL20 (NCIMB 30153) and *Bifidobacterium animalis* subsp. *lactis* CUL34 (NCIMB 30172) on a base of microcrystalline cellulose at a total of 5 × 10^10^ colony forming units (cfu) per capsule. The placebo product was capsules of microcrystalline cellulose and was identical in appearance to the active product. All products were prepared by Cultech Ltd, Port Talbot, UK and packed into induction-sealed high-density polyethylene pots and stored at 4–8 °C at the trial site; participants were instructed to refrigerate the supplement throughout the study.

### Intervention

One capsule was taken daily for 6 months (180 days). Participants were asked to consume the supplement with food (with or without a cool drink) at any time of the day and to avoid consumption within 2 hours of any antibiotic intake. Participants were asked to maintain their normal diet and lifestyle throughout the study while avoiding the consumption of other probiotic supplements. Participants were provided with pots containing 93 capsules at baseline and 3 months and unused capsules were collected at 3 months and 6 months for compliance monitoring and enumeration of viable bacteria; no deterioration in the product occurred during the intervention period (data not shown).

### Outcomes

Primary outcomes were changes from baseline in body weight, waist circumference (WC), blood pressure (BP) and plasma lipid profile (total cholesterol (TC), high-density lipoprotein cholesterol (HDL-C), low-density lipoprotein cholesterol (LDL-C) and triglycerides (TG)) in the total population. Secondary outcomes were changes from baseline in plasma CRP level, diversity of the faecal microbiota of volunteers, QoL (as measured by QoL questionnaire (QoLQ), Supplementary Fig. S1) and the incidence of URTI in the total population. Changes in body weight and plasma lipids in a stratified study population were also assessed.

### Data and sample collection

**Figure 1:**
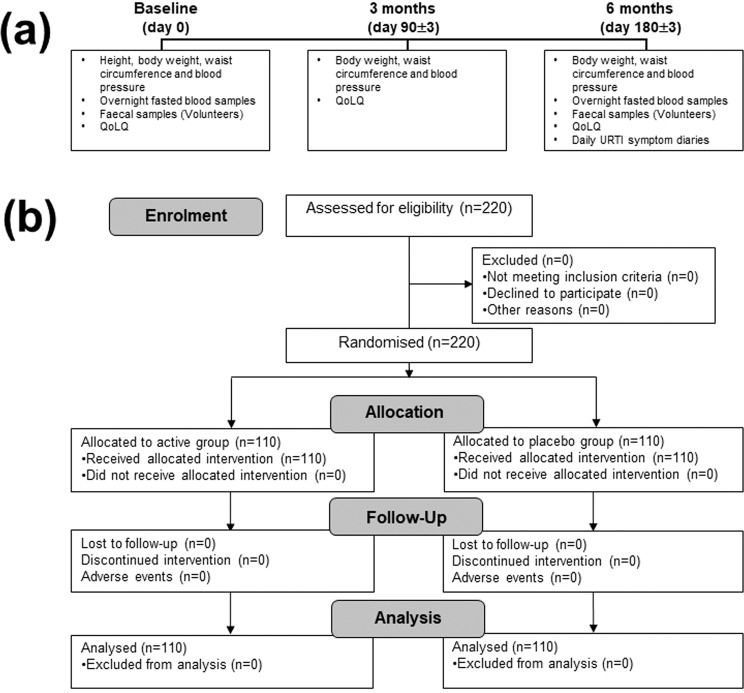
(**a**) Scheme of sample/data collection and (**b**) Flow diagram of the study. QoLQ, quality of life questionnaire; URTI, upper respiratory tract infection.

The schedule of data and sample collection is shown in Fig. 1a. Physiological measurements were taken at each visit. Overnight fasted blood samples were taken at baseline and 6 months. Participants were asked to complete daily diaries monitoring URTI symptoms^14^ throughout the duration of the intervention period and QoLQ were completed (Supplementary Fig. S1) at each visit. Participants volunteering to provide faecal samples used faecal collection kits for sample collection/transport.

### Physiological measurements

Body weights were recorded using a calibrated column scale (Seca 709, Hamburg, Germany) after the removal of shoes and jackets. Waist circumference was measured 2 fingers below the umbilicus and seated blood pressure was measured after 5 minutes respite using a calibrated blood pressure monitor (Omron, Kyoto, Japan). Height was measured after the removal of shoes. Efforts were made to ensure the time of day when measurements were taken was standardised for each participant.

### Processing and analysis of blood plasma

Overnight fasted bloods were collected into heparinised tubes and plasma separated by centrifugation (2,000 × g, 10 mins) and aliquoted within 1.5 hours of collection (stored at 4–8 °C). One aliquot was used to measure TC, HDL-C, LDL-C, TG and CRP immediately (Architect System clinical analyser^®^, Abbot Laboratories, Illinois, USA) and remaining plasma was stored at −80 °C until required. Levels of small dense (sd)LDL-C were determined using the sLDL-C-EX “SEIKEN” assay (Randox Laboratories Ltd, UK).

### Processing and analysis of faecal samples

Eighty volunteers provided faecal samples at baseline but at 6 months only 64/80 returned samples. Samples were stored refrigerated in anaerobic containers for no more than 48 h prior to storage at −80 °C pending analysis. Genomic DNA was extracted using the QIAamp^®^ Fast DNA Stool Mini Kit (Qiagen, UK) in accordance with manufacturer’s instructions in conjunction with a cell lysis step using Matrix Lysing B tubes (MP Biomedicals, USA) and a FastPrep^®^−24 bead beater (3 × 30 s cycles (5 m/s) with 5 minute intervals). Sample libraries were prepared using the Illumina 16S Metagenomic Sequencing Library Preparation Protocol with slight modifications; primers targeting *Bifidobacteria* species were included during amplification of the V1-V2 regions of the 16S rRNA as described elsewhere^15^ and PCR reactions were cleaned and normalised using the SequalPrep Normalization Plate Kit (Life Technologies, Paisley, UK). Sample libraries were then quantified using the NEBNext Library Quant Kit for Illumina (New England Biolabs, Hitchin, UK) and sequencing was performed on an Illumina MiSeq platform (Illumina Inc., Saffron Walden, UK) using the MiSeq Reagent Kit v3 (Illumina Inc) and paired-end 300 base pair (bp) chemistry. Negative and positive control reactions were included during sequencing that contained no template DNA or DNA from a reference bacterial community respectively.

SeqPrep C++ software was used to join the paired-end R1 and R2 reads. QIIME 1.9^16^ was used to process joined reads for quality-filtering with the following parameters: i) maximum of three consecutive base calls with Phred <19 (1 error in base calling in 100 bp), ii) a minimum read length including consecutive high-quality base calls (Phred > 19) and iii) no “n”-bases allowed. Quality-filtered reads were then aligned against the SILVA^17^ closed-reference database 123 release, with 97% cluster identity. To reduce the effect of uneven sampling and sequencing, each sample library was rarefied to the smallest library size.

### Data management and statistical analysis

Analysis of study outcomes was performed on an intention-to-treat basis. Outcome variables with measurements at 3 and 6 months were analysed using a linear mixed model (LMM) that included treatment, time, interaction between treatment and time as fixed effects, baseline measurement as a covariate, and subject as random effect. The treatment difference at each time point with 95% confidence intervals (CI) from t-test was calculated from the LMM. Outcome variables with only measurement at 6 months were analysed using a generalized linear model (GLM) that included treatment as the only predictor, and baseline measurement as a covariate, from which the treatment difference at 6 months with 95% CI was calculated.

Incidence rates were calculated from daily diaries by dividing the number of episodes of each symptom (coughing, runny nose, blocked nose, sore throat, headache, earache, muscle pain, chest wheeze and itchy eyes) or antibiotic usage by the number of days in the study and are expressed per 100 person days. Each episode comprised a continuous sequence of symptoms or antibiotic usage and was separated from another episode by a minimum of 24 h. Incidence rate ratios (IRR) were calculated using a GLM with Poisson distribution and log link function.

Covariate adjusted analyses within the LMM/GLM framework as described above were performed on all outcomes with age, gender and BMI as covariates. Where appropriate, subgroup analysis was performed by gender, age, BMI and TC level at baseline. Values of *p* were considered statistically significant when less than 0.05. Continuous variables were summarised using mean ± standard deviation (SD). Data analyses were performed using SAS^®^ version 9.4 (SAS Institute Inc., Cary, NC, USA).

### Statistical analysis of faecal microbiota

Statistical analysis of faecal next generation sequencing data was performed by calculating the unique number of operational taxonomic units (OTU) and indices of alpha-diversity (Chao1 and Shannon) and beta-diversity (weighted Unifrac) using QIIME 1.9. OTUs with less than 15 counts in at least two samples were removed from analysis. Differences in the alpha-diversity indices were tested using a mixed-effects linear model, implemented in the lmer function of the lme4 R package, with randomisation (active or placebo), time-point (baseline or 6 months), age, gender and BMI as fixed effects and each participant as a random effect. Two-sample comparisons were performed using either the t-test or the non-parametric Wilcoxon-Mann-Whitney test according to the normality distribution of the data. Between-group differences in beta-diversity were tested with the permutational analysis of variance (PERMANOVA), using the Adonis function of the Vegan R package. The assumption of homogeneity of dispersion amongst groups, required by PERMANOVA, was tested using the betadisper function in the Vegan R package.

## Results

### Recruitment

Two hundred and twenty participants were recruited to the study which took place between July 2017 and January 2018. There were no drop-outs, exclusions or adverse events in either arm of the study (Fig. 1b). Compliance to the intervention was greater than 99% in both arms of the study (as defined by number of returned capsules). Over the duration of the study, 12.7% of participants in the active group and 10.9% of participants in the placebo group reported antibiotic usage. Baseline demographics of the participants are shown in Table 1.

**Table 1:** Demographic and baseline characteristics of total study population.

	Active (N = 110)		Placebo (N = 110)	
	Mean	SD	Mean	SD
Study Demographics				
Age (years)	45.30	10.20	46.52	9.93
Males (n (%))	44 (40.0%)		43 (39.1%)	
Females (n (%))	66 (60.0%)		67 (60.9%)	
Statin usage (n (%))	1 (0.91%)		2 (1.81%)	
Physiological measurements				
Body weight (kg)	85.17	13.28	83.97	11.68
Height (m)	1.71	0.09	1.70	0.09
BMI (kg/m^2^)	29.14	2.73	28.97	2.86
WC (cm)	100.20	9.04	99.52	8.32
WtHR	0.59	0.04	0.59	0.05
SBP (mmHg)	128.64	12.73	130.41	5.76
DBP (mmHg)	79.05	5.29	78.93	5.40
Plasma biochemistry				
TC (mmol/L)	5.26	1.11	5.38	1.19
HDL-C (mmol/L)	1.37	0.33	1.33	0.32
LDL-C (mmol/L)	3.22	0.96	3.26	0.99
TG (mmol/L)	1.47	0.99	1.80	1.67
CRP (mg/L)	4.26	7.75	3.14	3.95
QoLQ Score				
General Wellness	7.65	1.87	7.75	1.80
State of health	7.97	1.74	8.02	1.74
State of energy	7.65	1.72	7.75	1.83
State of mood	7.82	1.94	7.81	1.77
Sleep quality	7.66	2.15	7.88	1.99

The data represents the mean ± standard deviation (SD) of 110 participants in each group. The number of participants (n) that were male or female or taking statin in each group are expressed as a percentage of the total group size.

### Physiological measurements

**Figure 2:**
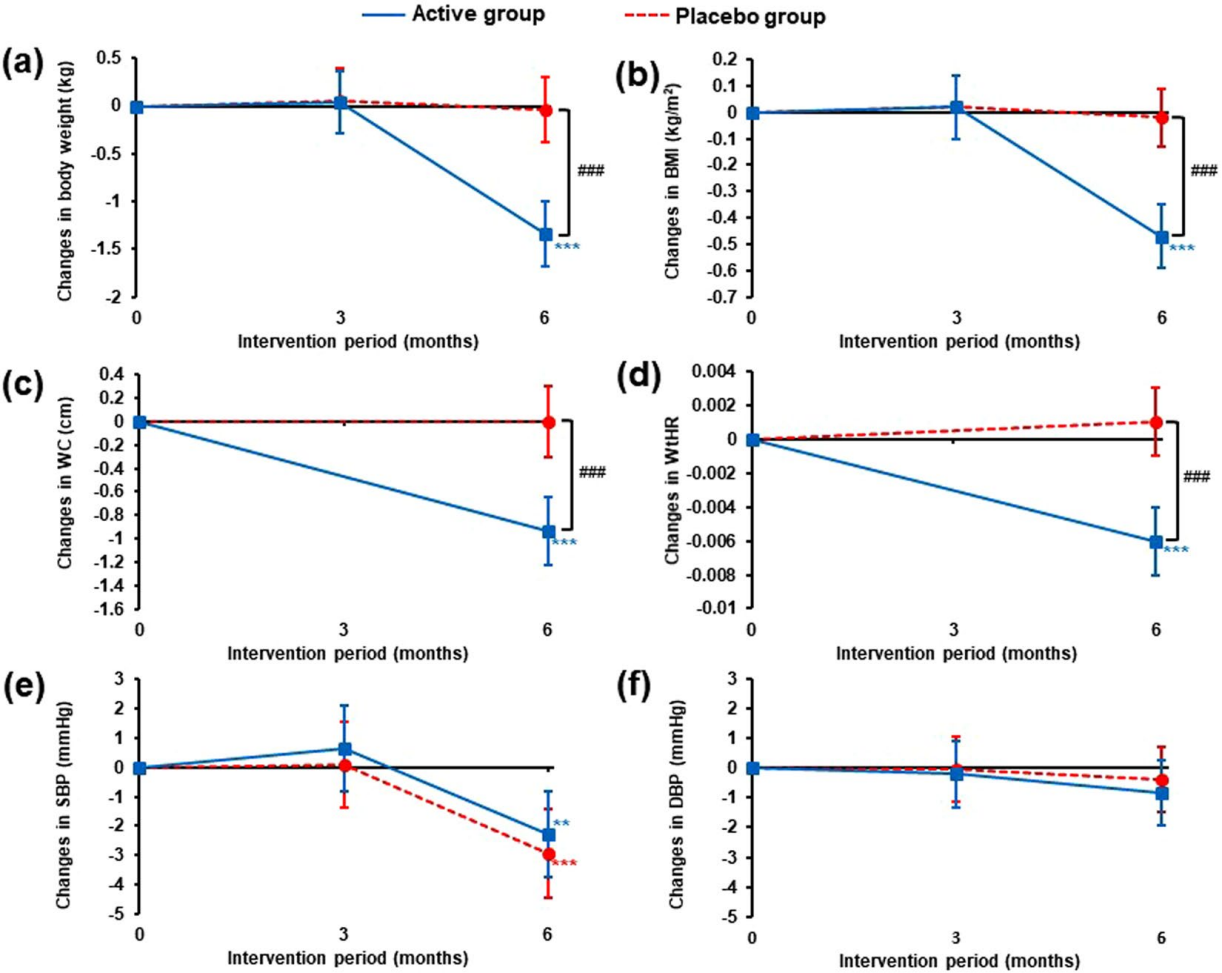
Changes from baseline in (**a**) body weight, (**b**) BMI, (**c**) WC, (**d**) WtHR, (**e**) SBP and (**f**) DBP over the duration of the intervention period. Data is presented as mean change from baseline (110 participants per group) with 95% CIs and *p* values were calculated using a LMM. For within group comparisons (vs baseline): **p ≤ 0.01 and ***p ≤ 0.001. For between group comparisons (active vs placebo): ^###^*p* ≤ 0.001. BMI, body mass index; WC, waist circumference; WtHR, waist-to-height ratio; SBP, systolic blood pressure; DBP, diastolic blood pressure.

Changes in body weight, BMI, WC, WtHR and blood pressure from baseline to 6 months are shown in Fig. 2 (detailed data presented in Supplementary Table S1). Significant between group differences at 6 months favouring probiotic supplementation were seen for body weight with a 1.5% weight reduction (-1.30 kg, *p* < 0.0001, Fig. 2a); significant weight loss occurred in the probiotic group (−1.34 kg, *p* < 0.0001) with no significant change in the placebo group. Significant between group reductions in response to the probiotic were seen for BMI (−1.5%, *p* < 0.0001, Fig. 2b), WC (−0.9%, *p* < 0.0001, Fig. 2c) and WtHR (−1.2%, *p* < 0.0001, Fig. 2d). Decreases in systolic blood pressure (SBP) were observed at 6 months in both groups (Active; −1.8%, *p* = 0.0026, Placebo; −2.2%, *p* < 0.0001, Fig. 2e) with no significant between group differences.

**Figure 3:**
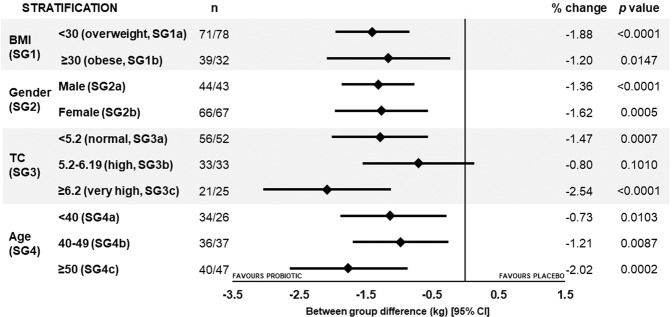
Forest plot of between group changes in body weight in the stratified subgroups at 6 months. Data is presented as mean change with 95% CIs and *p* values calculated using a LMM. n, number of participants (active/placebo); TC, total cholesterol; CI, confidence interval.

On the basis of population size, the opportunity for stratification of this population was explored and subgroup analysis of body weight was conducted: Subgroup 1 (SG1) based on clinically relevant values for overweight (BMI < 30 kg/m^2^, n = 149, SG1a) and obese (BMI ≥ 30 kg/m^2^, n = 71, SG1b); SG2 male (n = 87, SG2a) and female (n = 133, SG2b); SG3 based on clinical relevant values of normal TC levels (<5.2 mmol/L, n = 108, SG3a), high TC levels (5.2–6.19 mmol/L, n = 66, SG3b) and very high TC levels (≥6.2 mmol/L, n = 46, SG3c); SG4 based on age: SG4a <40 years, n = 60; SG4b 40–49 years, n = 73 and SG4c ≥ 50 years, n = 87. Significant between group differences in body weight in favour of the probiotic were observed for all subgroups after 6 months intervention (Fig. 3; detailed data presented in Supplementary Table S2).

For SG1, weight loss from baseline was greater in the overweight participants (BMI <30 kg/m^2^) supplemented with the Lab4P probiotic (−1.9%, −1.5 kg, SG1a) compared to the obese participants (−1.2%, −1.06 kg, SG1b) whilst body weight changes for both the placebo overweight and obese groups were <0.1%.

Lab4P supplementation resulted in 1.6% weight loss in women (−1.32 kg, SG2b) and 1.4% in men (−1.36 kg, SG2a), with no changes in either placebo group.

In the cholesterol based SGs, significant between group probiotic-mediated weight loss was observed in the normal and very high basal cholesterol subgroups. Significant weight loss occurred in the probiotic supplemented groups but the responses amongst the placebo groups were inconsistent with trends towards significant weight gain in the SG3c placebo group. The very high cholesterol probiotic group lost 1.47 kg (SG3c). Significant weight loss occurred in the probiotic receiving participants in sub-groups SG3a and SG3b.

Similarly, within the age stratifications (SG4) significant between group weight loss occurred in all subgroups favouring the probiotic with the greatest weight loss in the probiotic supplemented over 50 year olds (−1.72 kg).

### Plasma biochemistry

Table 2 shows no significant between group changes in plasma biochemistry for the total study population at 6 months. TC levels were unchanged in the total study population but LDL-C levels showed a 2.7% increase from baseline in both the active (0.087 mmol/L, *p* = 0.0667) and the placebo group (0.088 mmol/L, *p* = 0.0618). There were no significant between group differences or changes in levels of HDL-C, TG or CRP levels.

**Table 2:** Changes in plasma lipids and CRP from baseline in the total population at 6 months.

Outcome	Group	Difference (95% CI)	% change	*p* value
TC (mmol/L)	Between	0.016 (−0.15, 0.18)	0.29	0.8480
	Active	−0.009 (−0.12, 0.11)	−0.17	0.8809
	Placebo	−0.025 (−0.14, 0.09)	−0.46	0.6738
HDL-C (mmol/L)	Between	−0.014 (−0.70, 0.04)	−0.99	0.6350
	Active	0.005 (−0.03, 0.04)	0.36	0.8094
	Placebo	0.018 (−0.02, 0.06)	1.35	0.3614
LDL-C (mmol/L)	Between	−0.002 (−0.13, 0.13)	0.00	0.9806
	Active	0.087 (−0.01, 0.18)	2.70	0.0667
	Placebo	0.088 (0.00, 0.18)	2.70	0.0618
TG (mmol/L)	Between	0.153 (−0.10, 0.41)	8.69	0.2393
	Active	0.020 (−0.16, 0.20)	1.36	0.8326
	Placebo	−0.132 (−0.31, 0.05)	−7.33	0.1480
CRP (mg/L)	Between	−0.283 (−1.37, 0.81)	−7.35	0.6092
	Active	−0.199 (−0.97, 0.57)	−4.67	0.6100
	Placebo	0.084 (−0.68, 0.85)	2.68	0.8302

Data is presented as mean change (110 participants per group) with 95% CIs and *p* values calculated using a GLM. CI, confidence interval; TC, total cholesterol; HDL-C, high-density lipoprotein cholesterol; LDL-C, low-density lipoprotein cholesterol, TG, triglycerides; CRP, C-reactive protein.

**Figure 4:**
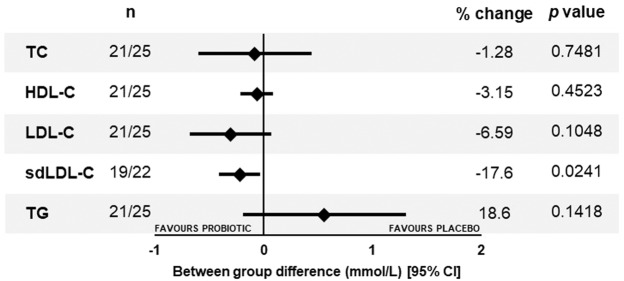
Forest plot of between group changes in plasma biochemistry in SG3c (TC ≥ 6.2 mmol/L) at 6 months. Data is presented as mean change with 95% CIs and *p* values were calculated using a GLM. n, number of participants (active/placebo); TC, total cholesterol; HDL-C, high-density lipoprotein cholesterol; LDL-C, low-density lipoprotein cholesterol; sdLDL-C, small dense LDL-C, TG, triglycerides; CI, confidence interval.

The results for the plasma samples collected from the participants in the SG3c subgroup are shown in Fig. 4 (detailed data presented in Supplementary Table S3) and indicated significant between group differences (−17.6%, −0.220 mmol/L, *p* = 0.0204) in the levels sdLDL-C as a result of a significant reduction of 15.2%, (*p* = 0.0090) in the active group and marginal increase in the placebo group. There was a between group reduction in plasma LDL-C levels (−0.305 mmol/L, *p* = 0.1048) favouring the probiotic that resulted from a significant 8.7% reduction from baseline in the Lab4P group (−0.396 mmol/L, *p* = 0.0055) and no significant change in the placebo group. TC, HDL and TG did not differ between groups.

### Faecal microbiota

**Figure 5:**
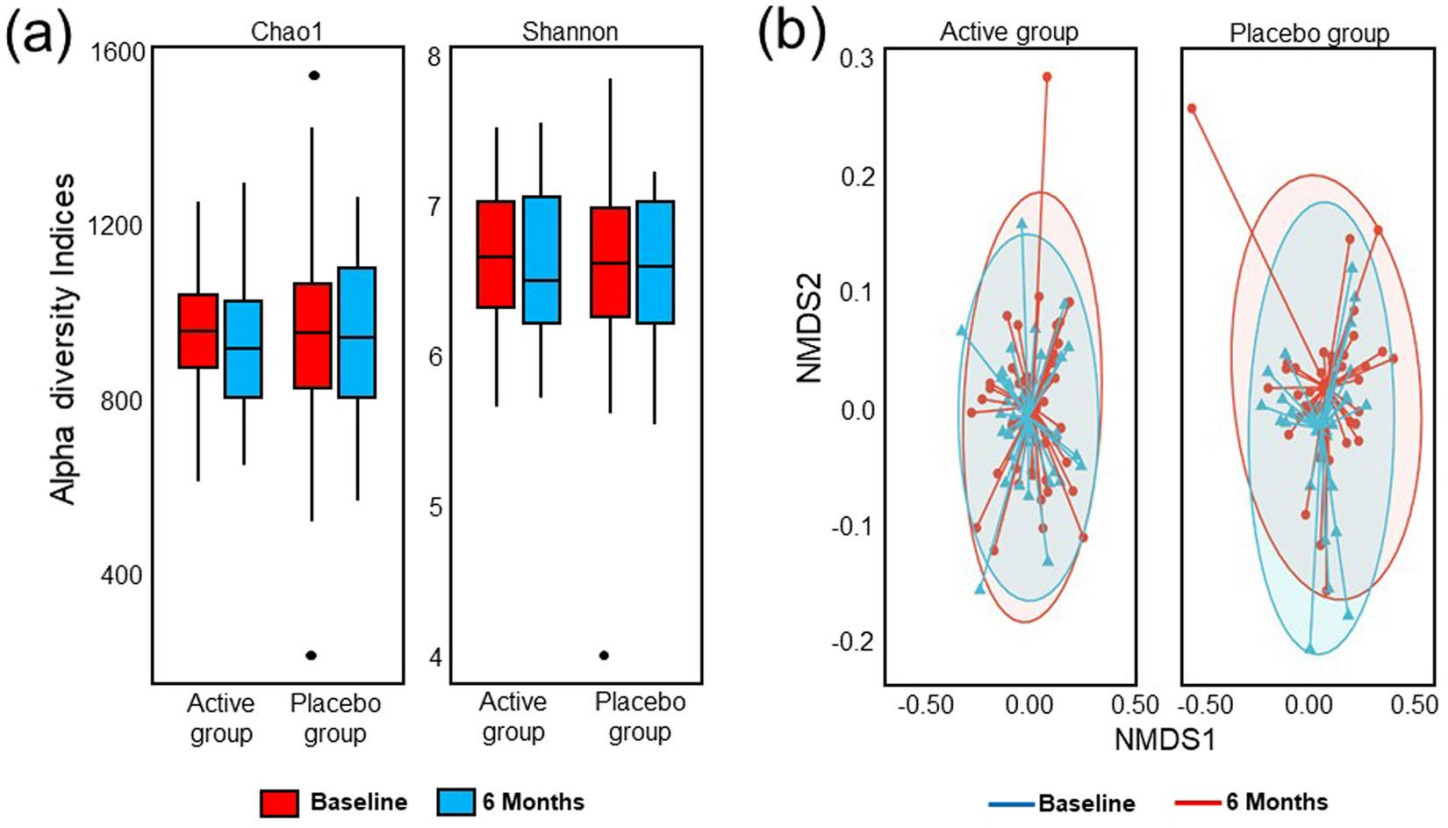
Diversity within the faecal microbiota of the active and placebo groups over the duration of the study. (**a**) Box-and-whisker plot showing the Chao1 and Shannon diversity (alpha-diversity) and (**b**) a non-metric dimensional scaling (NMDS) plot showing the weighted unifrac (beta-diversity) of the active group and placebo group at baseline and 6 months were generated and differences were assessed with PERMANOVA; statistical outliers are represented as black dots and no significant changes were observed. Ellipses represent the 95% confidence interval. The data represents 80 samples at baseline (43 active and 37 placebo) and 64 samples at 6 months (35 active and 29 placebo) from which a total of 3,522,472 reads (mean reads per sample = 24,461 ± 7,399) were retained after quality filtering and 7,075 unique OTUs were identified and quantified (0.1 non-zero values fraction). 11,001 reads per sample were obtained after rarefaction to the smallest library size. Filtering of the low-abundant OTUs retained 2,047 OTUs that were grouped into 11 phyla and 205 genera.

From the analysis of the 144 faecal samples provided by volunteers, a total of 3,522,472 reads (mean reads/sample = 24,461 ± 7,399) were obtained after quality filtering resulting in 7,075 unique operational taxonomic units (OTUs). After rarefaction to the smallest library size 11,001 reads per sample resulted in 2,047 OTUs that were grouped into 11 phyla and 205 genera. Fig. 5a,b indicate that there were no significant differences in either alpha- or beta-diversity between the probiotic and placebo groups at baseline or 6 months. Of the participants providing faecal samples, antibiotic use was reported by 10% participants in the active group and 10% participants in the placebo group.

### Quality of Life

In the total population, supplementation with the probiotic improved General Wellness scores by 6.3% (*p* = 0.0091) and 5.6% (*p* = 0.0261) at 3 months and 6 months respectively (Table 3) compared to placebo. For State of Health, State of Energy, and State of Mood, there were also significant between group benefits at 3 months of 5.8% (0.45, *p* = 0.013), 5.5% (0.42, *p* = 0.0316) and 5.1% (0.40, *p* = 0.0337) respectively but no differences were observed at 6 months. Sleep quality did not differ between groups. Within group differences in QoL scores are provided in Supplementary Table S4.

**Table 3:** Changes from baseline in quality of life questionnaire (QoLQ) scores in the total study population.

Outcome	3 months			6 months		
	Difference (95% CI)	% change	*p* value	Difference (95% CI)	% change	*p* value
General Wellness	0.49 (0.12, 0.85)	6.34	0.0091	0.42 (0.05, 0.78)	5.56	0.0261
State of Health	0.45 (0.10, 0.81)	5.78	0.013	0.24 (−0.12, 0.59)	3.03	0.1939
State of Energy	0.42 (0.04, 0.81)	5.53	0.0316	0.09 (−0.30, 0.47)	1.25	0.6610
State of Mood	0.40 (0.03, 0.76)	5.11	0.0337	0.08 (−0.29, 0.44)	1.01	0.6747
Sleep Quality	0.25 (−0.22, 0.71)	3.20	0.2997	−0.22 (−0.68, 0.25)	−2.56	0.3569

Data is presented as mean changes (110 participants per group) with 95% CIs and p values were calculated using a LMM. CI, confidence interval.

### Incidence rate ratio of URTI symptoms

For the total population, the probiotic reduced the incidence of URTI symptoms by 40% (*p* < 0.0001, Table 4) and analysis of individual URTI symptoms indicates significant reductions in sneezing (−46%, *p* < 0.0001), coughing (−33%, *p* = 0.0073) and blocked nose (−28%, *p* = 0.0181) together with IRR reductions for headache (−32%, *p* < 0.0001), earache (−52%, *p* = 0.0173) and muscle ache (−33%, *p* = 0.0225) compared to placebo. Twenty six participants reported antibiotic usage during the intervention period. Distribution of these participants was comparable between the groups with 14 in the active and 12 in the placebo.

**Table 4:** Incidence rates of URTI in the total study population.

	Incidence Rate (per 100 person days)		*p* value
	Active	Placebo	
**URTI Symptoms**	1.30	2.16	
*IRR (95% CI)*	0.60 (0.52, 0.70)		<0.0001
**Individual URTI symptoms**			
Sneezing	0.71	1.31	
*IRR (95% CI)*	0.54 (0.44, 0.66)		<0.0001
Cough	0.38	0.57	
*IRR (95% CI)*	0.67 (0.50, 0.90)		0.0073
Runny nose	0.46	0.52	
*IRR (95% CI)*	0.89 (0.67, 1.18)		0.4268
Blocked nose	0.46	0.64	
*IRR (95% CI)*	0.72 (0.55, 0.95)		0.0181
Sore Throat	0.25	0.31	
*IRR (95% CI)*	0.80 (0.55, 1.17)		0.2512
**Other symptoms**			
Headache	1.08	1.58	
*IRR (95% CI)*	0.68 (0.58, 0.81)		<0.0001
Earache	0.08	0.17	
*IRR (95% CI)*	0.48 (0.27, 0.88)		0.0173
Muscle pain	0.28	0.42	
*IRR (95% CI)*	0.67 (0.48, 0.95)		0.0225
Chest wheeze	0.09	0.15	
*IRR (95% CI)*	0.62 (0.34, 1.12)		0.1112
Itchy eyes	0.19	0.23	
*IRR (95% CI)*	0.80 (0.52, 1.24)		0.3218
**Antibiotic usage**	0.08	0.08	
*IRR (95% CI)*	0.94 (0.46, 1.89)		0.8552

Data is presented as incidence rate or incidence rate ratios (IRR) of 110 participants per group with 95% CIs and *p* values were calculated using a GLM with Poisson distribution and log link function. CI, confidence interval.

## Discussion

Supplementation of a free-living population of healthy overweight or obese individuals with the Lab4P probiotic resulted in significant reductions in body weight, BMI, waist circumference and waist-to-height ratio compared to the placebo group. Significant decreases in systolic blood pressure and significant decreases in LDL-C occurred in both groups.

The sample size of our study population afforded the opportunity for subgroup stratification with meaningful numbers. Probiotic-mediated weight loss was observed within subgroups with the overweight (SG1a, 1.9% loss), female (SG2b, 1.6% loss), hypercholesterolaemic (SG3c, 2.5% loss) and over 50 s (SG4c, 2.0% loss) groups presenting with the greatest weight decreases. No significant weight changes were observed in any of the placebo subgroups but a tendency towards significant weight loss was seen in the high basal cholesterol subgroup (SG3b) and a tendency towards significant weight gain was observed in the very high basal cholesterol subgroup (SG3c).

Despite significant changes in the anthropometric characteristics, no changes in plasma levels of TC, LDL-C, HDL-C, TG nor CRP were observed either between the groups or over the intervention period for the total study population. But in the very high cholesterol sub-group SG3c, reductions in plasma LDL-C were observed within the probiotic group and reductions in sdLDL-C were observed between groups favouring the probiotic supplementation.

Globally it is estimated that 1.9 billion adults are overweight with 650 million of these categorised as obese^1^. Our study is one of the first to report the impact of a multi-strain probiotic on weight loss in response to 6-months supplementation in a cohort of healthy, overweight and obese, free-living (no dietary or lifestyle restrictions) subjects. More weight loss occurred in the overweight subjects (SG1a) compared with the obese (SG1b) receiving probiotics agreeing with the meta-analysis findings of Koutnikova and colleagues of greater weight loss in probiotic supplemented overweight participants than obese^10^. No weight loss was observed at the midpoint (3 months) of our study as has been shown in a number of short-term probiotic studies in free-living overweight/obese subjects^18–20^. The association between longer intervention periods and weight loss has been seen in other meta-analyses^21–24^. Supplementation with Lab4P had a greater impact on females (SG2b), who lost more weight than males (SG2a). Similar observations have been made in diet-restricted probiotic intervention studies and are thought to reflect differences in gut microbiota composition and/or energy utilisation between sexes^25,26^.

In the most recent meta-analyses detailing probiotics and weight loss, body weight reductions up to 1.05 kg and BMI reductions up to 0.55 kg/m^2^ have been reported for overweight/obese subjects^10,22,27^. Studies focusing on free-living overweight and/or obese participants present variable outcomes^18–20,28–34^ with many performed in Asian populations^18,29–34^. In overweight and/or obese Japanese^29–31^ or Korean subjects^32–34^ significant reductions in body weight and/or BMI (0.6 to 1.24 kg; 0.23 to 0.45 kg/m^2^, respectively) have been reported in 12-week probiotic studies. No changes were observed in the body weights of overweight/obese Finnish participants supplemented with *Bifidobacterium animalis* ssp*. lactis* 420, for 24 weeks^28^ nor in overweight Japanese subjects supplemented with *Bifidobacterium breve* B3 for 12 weeks^18^. In a 6-week study with Polish participants (BMI ≈25 kg/m^2^) supplemented with *Lactobacillus plantarum* 299 v, no significant changes in weight/BMI^19^ were reported nor in a 4 week study with lean (BMI <25 kg/m^2^) and obese (BMI ≥35 kg/m^2^) German participants supplemented with *Lactobacillus reuteri* SD5865^20^. Depommier *et al*. (2019) supplemented obese individuals with metabolic disease with a gut-derived strain of *Akkermansia muciniphila*, which is an organism believed to be linked to weight management, and they observed non-significant body weight reductions after 3 months^35^.

Waist circumference and WtHR ratio are used in clinical practice as markers of visceral adiposity^36^, closely associated with diabetes and CVD^37^. Supplementation with Lab4P resulted in significant reductions in WC and WtHR for the total population and similar outcomes have been reported in a number of probiotic intervention studies^18,29–31,33^ with changes sometimes occurring in the absence of body weight reduction^18,28^.

The proposed mechanisms of action for probiotic-mediated weight loss includes the modulation of the composition of the gut microbiota and the production of short chain fatty acids, the regulation of energy homeostasis and/or satiety, improved gut barrier function and the interruption of bile acid metabolism in the host^38^. We did not detect any gross changes in the faecal microbiota in response to the probiotic. Khalesi *et al*. (2019) observed that probiotic supplementation in healthy adults may not result in changes in the composition of the gut microbiota^11^.

It has been shown that the presence of bacteria with bile salt hydrolase (BSH) activity can mediate the deconjugation of bile acids that has been implicated in the prevention of weight gain^39^. The Lab4P consortium has previously been shown to possess bile salt hydrolase activity^12,13^ and in C57BL/6J mice fed a high fat diet with/without Lab4P, increased faecal deconjugated bile acid levels, reduced circulating cholesterol levels and reduced diet induced weight gain were observed in the Lab4P fed mice^13^. Bacterial BSH activity is also linked with the reduction of circulating cholesterol levels^40^.

In our study population, cholesterol and other plasma lipids were not significantly changed and support the observation that probiotic supplementation may have little impact in plasma lipids levels in healthy subjects^11^. However, in the hypercholesterolaemic SG3c subgroup (weight loss:1.47 kg), significant plasma LDL-C reductions of 0.4 mmol/L from baseline were detected in the probiotic group which supports the findings of our previous mouse studies with Lab4P^13^. A meta-analysis that assessed the impact of probiotics on lipid levels reported comparable changes in circulating LDL-C levels in hypercholesterolaemic subjects^41^. It has been estimated that LDL-C reductions in the region of 1 mmol/L can lead to a 23% reduction in the risk of a CVD-related event^42^.

One of the underlying causes of CVD is the incidence of atherosclerosis, an inflammatory disease of the vasculature driven by the accumulation of modified forms of LDL-C in artery walls^43^. LDL is heterogeneous molecule comprising a number of discrete particle subclasses that vary in size, density and cholesterol content and the small dense LDL-C (sdLDL-C) are considered highly atherogenic^44^. High circulating levels of sdLDL-C are linked to increased CVD risk (irrespective of LDL-C levels^45^) and an increased susceptibility to obesity and metabolic syndrome^46^. In this study, we recorded between group reductions of sdLDL-C in excess of 17% in hypercholesterolaemic participants (SG3c).

Obesity is also known to impact upon quality of life resulting from factors such as less ability to perform activities and early fatigue^47^, increased anxiety, depression and low self-esteem^48^. Using a modification of an existing validated QoL questionnaire^49^, the results indicated significant improvements in participant scores for general wellness, state of health/energy/mood but not sleep quality after 3 months Lab4P supplementation. At 6 months, the only difference was in general wellness. QoL improvements have been seen in a study with a probiotic/herbal formulation^50^ and Blissmer *et al*. (2006) demonstrated improvements in quality of life associated with weight loss^51^.

Excess body weight has been linked to immunological imbalances that can manifest as increased susceptibility to infections including URTIs^52^; such infections can impart a considerable socioeconomic burden^53^. Probiotics have been found to have a preventative effect on the incidence/severity of URTI^54^. Our group has shown reductions in the incidence of URTI symptoms (sneezing, coughing, blocked nose, runny nose and sore throat) in children receiving probiotics^14^ and these observations have been supported by our *in vitro* evidence of probiotic-mediated immunomodulatory activity^55^. In the current study, there was significantly less sneezing, coughing and blocked nose reported by those participants receiving Lab4P compared to the placebo and the overall incidence ratio of URTI was reduced in the probiotic group. Lab4P supplementation also significantly reduced the incidence of headache, earache and muscle pain although antibiotic usage did not differ between groups.

Strengths of our study include the large population size allowing subgroup analysis although it should be noted that subgroup analysis could have been influenced by the randomisation although similar numbers of active and placebo participants were present in each sub group. Another strength is the duration of the intervention period and the unadjusted lifestyle conditions. The limitations of our study include the lack of a formal power calculation due to the exploratory nature of the study, its geographical isolation (single centre) and also the free living nature of the participants (no dietary control). It is also possible that the inclusion of participants receiving antibiotics in our analyses may have influenced our findings although the incidence of antibiotic usage was low (≈10% in each arm of the study) and was evenly distributed between both groups. Further work will involve assessments of the microbiota of the individuals that received antibiotics.

In summary, this exploratory study has demonstrated that 6 months Lab4P supplementation at 50 billion cfu/day significantly reduced bodyweight, BMI, waist circumference and waist-to-height ratio in a free-living overweight/obese population with greater weight loss observed in the overweight and the female participants. Greatest weight loss together with decreases in small dense LDL-C level were observed in hypercholesterolaemic participants. Limited changes in response to supplementation, if any, were observed at 3 months suggesting that 6 months supplementation at this dosage of Lab4P was needed to effect a meaningful change. Improvements in other measures such as participant-perceived QoL and URTI symptoms highlight the holistic benefits of the Lab4P supplementation. Further adequately powered target studies are needed to confirm these findings and to assess the impact in a multi-centre study.

## Supplementary information

Supplementary Information.

## Data Availability

The datasets generated during and/or analysed during the current study are available from the corresponding author on reasonable request. Sequencing data generated during the current study is available for download from the NCBI genbank database (Bioproject ID: PRJNA606023, http://www.ncbi.nlm.nih.gov/bioproject/606023).
