# Supplementary material for: A randomised controlled study shows supplementation of overweight and obese adults with lactobacilli and bifidobacteria reduces bodyweight and improves well-being
Source: Sci Rep. 2020 Mar 6;10:4183. doi: 10.1038/s41598-020-60991-7 (PMC7060206; doi:10.1038/s41598-020-60991-7)
Supplement: Supplementary file 1 — Supplementary Information. [file 41598_2020_60991_MOESM1_ESM.docx]

**A randomised controlled study shows supplementation of overweight and obese adults with lactobacilli and bifidobacteria reduces bodyweight and improves well-being**

D. R. Michael, A. A. Jack, G. Masetti, T. S. Davies, K. E. Loxley, J. Kerry-Smith, J. F. Plummer, J. R. Marchesi, B. H. Mullish, J. A. K. McDonald, T. R. Hughes, D. Wang, I. Garaiova,  Z. Paduchová, J. Muchová, M. A. Good and S. F. Plummer

**SUPPLEMNTARY DATA**

**Supplementary Figure S1. Quality of Life Questionnaire (QoLQ)**

| **Participant number:** |  | | | | | | |  |  |  |  |  |  |  |
| --- | --- | --- | --- | --- | --- | --- | --- | --- | --- | --- | --- | --- | --- | --- |
| **Date (dd/mm/yy):** |  | | | | | | |  |  |  |  |  |  |  |
| **Stage of trial**  **(please circle as appropriate)** | 1 | | 2 | | 3 | | |  |  |  |  |  |  |  |
|  | | | | | | | | | | | | | | |
| **Over the period of the last 3 months, how would you rate your:**  (please circle as appropriate with a score of 0 being very poor and a score of 10 being very good) | | | | | | | | | | | | | | |
| **General wellness** | 0 | 1 | | 2 | | 3 | 4 | | 5 | 6 | 7 | 8 | 9 | 10 |
| **State of health** | 0 | 1 | | 2 | | 3 | 4 | | 5 | 6 | 7 | 8 | 9 | 10 |
| **State of energy** | 0 | 1 | | 2 | | 3 | 4 | | 5 | 6 | 7 | 8 | 9 | 10 |
| **State of mood** | 0 | 1 | | 2 | | 3 | 4 | | 5 | 6 | 7 | 8 | 9 | 10 |
| **Sleep quality** | 0 | 1 | | 2 | | 3 | 4 | | 5 | 6 | 7 | 8 | 9 | 10 |

**Supplementary Table S1: Physiological changes from baseline in the total study population**

| **Outcome** | **Group(s)** | **3 months** | |  | **6 months** | | |
| --- | --- | --- | --- | --- | --- | --- | --- |
|  |  | **% change** | ***p* value** |  | **Difference**  **(95% CI)** | **% change** | ***p* value** |
| **Weight (kg)** | Between | -0.01 | 0.9843 |  | -1.30 (-1.77, -0.83) | -1.53 | <0.0001 |
|  | Active | 0.05 | 0.7994 |  | -1.34 (-1.68, -1.01) | -1.57 | <0.0001 |
|  | Placebo | 0.06 | 0.7779 |  | -0.04 (-0.38, 0.29) | -0.05 | 0.7994 |
| **BMI (kg/m^2^)** | Between | 0.00 | 0.9956 |  | -0.45 (-0.62, -0.28) | -1.54 | <0.0001 |
|  | Active | 0.07 | 0.7448 |  | -0.47 (-0.59, -0.35) | -1.61 | <0.0001 |
|  | Placebo | 0.07 | 0.7390 |  | -0.02 (-0.13, 0.10) | -0.07 | 0.7736 |
| **WC**  **(cm)** | Between | NM | |  | -0.94 (-1.35, -0.52) | -0.94 | <0.0001 |
|  | Active | NM | |  | -0.93 (-1.22, -0.63) | -0.93 | <0.0001 |
|  | Placebo | NM | |  | 0.01 (-0.29, 0.30) | 0.01 | 0.9549 |
| **WtHR** | Between | NM | |  | -0.006 (-0.009, -0.003) | -1.19 | <0.0001 |
|  | Active | NM | |  | -0.006 (-0.008, -0.004) | -1.02 | <0.0001 |
|  | Placebo | NM | |  | 0.001 (-0.001, 0.003) | 0.17 | 0.5347 |
| **SBP (mmHg)** | Between | 0.43 | 0.6035 |  | 0.69 (-1.40, 2.77) | 0.47 | 0.5168 |
|  | Active | 0.50 | 0.3897 |  | -2.28 (-3.75, -0.81) | -1.77 | 0.0026 |
|  | Placebo | 0.07 | 0.9002 |  | -2.92 (-4.43, -1.49) | -2.24 | <0.0001 |
| **DBP (mmHg)** | Between | -0.21 | 0.8283 |  | -0.44 (-2.00, 1.13) | -0.54 | 0.5389 |
|  | Active | -0.25 | 0.7165 |  | -0.82 (-1.93, 0.29) | -1.04 | 0.1451 |
|  | Placebo | -0.04 | 0.9550 |  | -0.39 (-1.50, 0.72) | -0.49 | 0.4930 |

Data is presented as mean change (110 participants per group) with 95% confidence intervals (CIs) and *p* values calculated using a linear mixed model (LMM).

*Abbreviations:* BMI, body mass index; WC, waist circumference; WtHR, waist to height ratio; SBP, systolic blood pressure; DBP, diastolic blood pressure; NM, not measured

Supplementary Table S2: Body weight changes from baseline in the stratified subgroups at 6 months.

| Stratification | | **Group(s)** | **n** | **Baseline Mean (SD)** | **6 Months** | | |
| --- | --- | --- | --- | --- | --- | --- | --- |
|  |  |  |  |  | **Difference**  **(95% CI)** | **%** | ***p* value** |
| **BMI**  **(SG1)** | **<30**  **(*SG1a*)** | Between | 71/78 |  | -1.40 (-1.95, -0.84) | -1.88 | <0.0001 |
|  |  | Active | 71 | 79.14(10.05) | -1.50 (-1.90, -1.10) | -1.90 | <0.0001 |
|  |  | Placebo | 78 | 80.03(9.74) | -0.10 (-0.48, 0.28) | -0.01 | 0.6080 |
|  |  |  |  |  |  |  |  |
|  | **≥30**  **(*SG1b*)** | Between | 39/32 |  | -1.16 (-2.08, -0.23) | -1.20 | 0.0147 |
|  |  | Active | 39 | 96.15(11.32) | -1.06 (-1.68, -0.45) | -1.10 | 0.0010 |
|  |  | Placebo | 32 | 93.58(10.46) | 0.09 (-0.59, 0.78) | 0.10 | 0.7863 |
| **Gender**  **(SG2)** | **Male**  **(*SG2a*)** | Between | 44/43 |  | -1.31 (-1.86, -0.76) | -1.36 | <0.0001 |
|  |  | Active | 44 | 96.50(10.16) | -1.36 (-1.74, -0.97) | -1.41 | <0.0001 |
|  |  | Placebo | 43 | 92.16(10.17) | -0.05 (-0.44, 0.34) | -0.05 | 0.7906 |
|  |  |  |  |  |  |  |  |
|  | **Female**  **(*SG2b*)** | Between | 66/67 |  | -1.26 (-1.96, -0.56) | -1.62 | 0.0005 |
|  |  | Active | 66 | 77.62(9.10) | -1.32 (-1.81, -0.82) | -1.70 | <0.0001 |
|  |  | Placebo | 67 | 78.71(9.36) | -0.06 (-0.55, 0.44) | -0.08 | 0.8259 |
| **TC**  **(SG3)** | **<5.2**  **(*SG3a*)** | Between | 56/52 |  | -1.28 (-2.01, -0.56) | -1.47 | 0.0007 |
|  |  | Active | 56 | 86.96(13.92) | -1.36 (-1.86, -0.86) | -1.56 | <0.0001 |
|  |  | Placebo | 52 | 85.48(12.09) | -0.08 (-0.60, 0.44) | -0.09 | 0.7717 |
|  |  |  |  |  |  |  |  |
|  | **5.2-6.19**  **(*SG3b*)** | Between | 33/33 |  | -0.70 (-1.54, 0.14) | -0.80 | 0.1010 |
|  |  | Active | 33 | 84.70(13.23) | -1.21 (-1.80, -0.62) | -1.43 | 0.0001 |
|  |  | Placebo | 33 | 81.42(12.29) | -0.51 (-1.11, 0.08) | -0.63 | 0.0866 |
|  |  |  |  |  |  |  |  |
|  | **≥6.2**  **(*SG3c*)** | Between | 21/25 |  | -2.08 (-3.04, -1.12) | -2.54 | <0.0001 |
|  |  | Active | 21 | 81.14(11.04) | -1.47 (-2.17, -0.76) | -1.81 | 0.0001 |
|  |  | Placebo | 25 | 84.18(9.66) | 0.61 (-0.04, 1.26) | 0.72 | 0.0637 |
| **Age**  **(SG4)** | **<40**  **(*SG4a*)** | Between | 34/26 |  | -1.13 (-1.99, -0.28) | -0.73 | 0.0103 |
|  |  | Active | 34 | 87.44(13.08) | -0.89 (-1.45, -0.33) | -1.02 | 0.0024 |
|  |  | Placebo | 26 | 84.54(10.38) | 0.24 (-0.40, 0.88) | -0.28 | 0.4546 |
|  |  |  |  |  |  |  |  |
|  | **40-49**  **(*SG4b*)** | Between | 36/37 |  | -0.97 (-1.69, -0.25) | -1.21 | 0.0087 |
|  |  | Active | 36 | 81.50(11.77) | -1.33 (-1.84, -0.82) | -1.63 | <0.0001 |
|  |  | Placebo | 37 | 84.57(13.35) | -0.36 (-0.86, 0.15) | -0.43 | 0.1630 |
|  |  |  |  |  |  |  |  |
|  | **≥50**  **(*SG4c*)** | Between | 40/47 |  | -1.76 (-2.64, -0.87) | -2.02 | 0.0002 |
|  |  | Active | 40 | 86.55(14.33) | -1.72 (-2.37, -1.08) | -1.99 | <0.0001 |
|  |  | Placebo | 47 | 83.18(11.13) | 0.03 (-0.56, 0.63) | 0.04 | 0.9171 |

Data is presented as mean change (110 participants per group) with 95% CIs and *p* values calculated using a generalized mixed model (GLM).

*Abbreviations:* n, number of participants; BMI, body mass index; WC, waist circumference; WtHR, waist to height ratio; SBP, systolic blood pressure; DBP, diastolic blood pressure; NM, not measured.

Supplementary Table S3: Changes from baseline in plasma biochemistry in SG3c (TC ≥6.2 mmol/L)

| Outcome | **Group(s)** | **n** | **Baseline Mean (SD)** | **SG3c: TC ≥6.2 mmol/L** | | |
| --- | --- | --- | --- | --- | --- | --- |
|  |  |  |  | **Difference (95% CI)** | **% change** | ***p* value** |
| **TC (mmol/L)** | Between | 21/25 |  | -0.083 (-0.60, 0.44) | -1.28 | 0.7481 |
|  | Active | 21 | 6.92(0.50) | -0.558 (-0.94, -0.18) | -8.06 | 0.0053 |
|  | Placebo | 25 | 7.00(0.87) | -0.475 (-0.83, -0.12) | -6.79 | 0.0092 |
| **HDL-C (mmol/L)** | Between | 21/25 |  | -0.056 (-0.21, 0.09) | -3.15 | 0.4523 |
|  | Active | 21 | 1.52(0.39) | -0.121 (-0.23, -0.01) | -7.96 | 0.0308 |
|  | Placebo | 25 | 1.33(0.35) | -0.064 (-0.16, 0.04) | -4.81 | 0.2002 |
| **LDL-C (mmol/L)** | Between | 21/25 |  | -0.305 (-0.68, 0.07) | -6.59 | 0.1048 |
|  | Active | 21 | 4.58(0.52) | -0.396 (-0.67, -0.12) | -8.65 | 0.0055 |
|  | Placebo | 25 | 4.43(0.94) | -0.091 (-0.34, 0.16) | -2.05 | 0.4694 |
| **sdLDL-C (mmol/L)** | Between | 19/22 |  | -0.220 (-0.41, -0.03) | -17.60 | 0.0241 |
|  | Active | 19 | 1.25(0.51) | -0.190 (-0.33, -0.05) | -15.20 | 0.0090 |
|  | Placebo | 22 | 1.29(0.53) | 0.031 (-0.100, 0.16) | 2.40 | 0.6272 |
| **TG (mmol/L)** | Between | 21/25 |  | 0.558 (-0.19, 1.31) | 18.60 | 0.1418 |
|  | Active | 21 | 1.89(1.32) | 0.049 (-0.49, 0.59) | 2.59 | 0.8570 |
|  | Placebo | 25 | 3.18(2.59) | -0.509 (-1.00, -0.01) | -16.01 | 0.0444 |

Data is presented as mean change with 95% CIs and *p* values were calculated using a GLM.

*Abbreviations:* n, number of participants; TC, total cholesterol; HDL-C, high-density lipoprotein cholesterol; LDL-C, low-density lipoprotein cholesterol; sdLDL-C, small dense LDL, TG, triglycerides; CRP, C-reactive protein.

**Supplementary Table S4: Changes from baseline in quality of life questionnaire (QoLQ) scores in the active and placebo groups of the total study population**

| Outcome | **Group(s)** | **3 months** | | |  | **6 months** | | |
| --- | --- | --- | --- | --- | --- | --- | --- | --- |
|  |  | **Difference (95% CI)** | **% change** | ***p* value** |  | **Difference (95% CI)** | **% change** | ***p* value** |
| **General Wellness** | Active | 0.89 (0.64, 1.15) | 11.63 | <0.0001 |  | 0.84 (0.58, 1.10) | 10.98 | <0.0001 |
|  | Placebo | 0.41 (0.15, 0.66) | 5.29 | 0.0022 |  | 0.42 (0.17, 0.68) | 5.42 | 0.0014 |
| **State of Health** | Active | 0.59 (0.33, 0.84) | 7.40 | <0.0001 |  | 0.48 (0.22, 0.73) | 6.02 | 0.0003 |
|  | Placebo | 0.13 (-0.12, 0.38) | 1.62 | 0.3065 |  | 0.24 (-0.01, 0.49) | 2.99 | 0.0622 |
| **State of Energy** | Active | 0.65 (0.38, 0.92) | 8.50 | <0.0001 |  | 0.52 (0.24, 0.79) | 6.80 | 0.0002 |
|  | Placebo | 0.23 (-0.04, 0.50) | 2.97 | 0.0969 |  | 0.43 (0.16, 0.70) | 5.55 | 0.0021 |
| **State of**  **Mood** | Active | 0.65 (0.39, 0.91) | 8.31 | <0.0001 |  | 0.67 (0.41, 0.92) | 8.57 | <0.0001 |
|  | Placebo | 0.25 (-0.01, 0.51) | 3.20 | 0.0559 |  | 0.59 (0.33, 0.85) | 7.55 | <0.0001 |
| **Sleep Quality** | Active | 0.41 (0.08, 0.74) | 5.35 | 0.0140 |  | 0.30 (-0.03, 0.62) | 3.92 | 0.0781 |
|  | Placebo | 0.17 (-0.16, 0.50) | 2.16 | 0.3150 |  | 0.51 (0.18, 0.84) | 6.47 | 0.0024 |

Data is presented as mean changes (110 participants per group) with 95% CIs and *p* values were calculated using a LMM.
